# Hospital antibiotic consumption—an interrupted time series analysis of the early and late phases of the COVID-19 pandemic in Poland, a retrospective study

**DOI:** 10.1007/s43440-023-00479-z

**Published:** 2023-04-05

**Authors:** Małgorzata Siewierska, Mateusz Gajda, Aleksandra Opalska, Michał Brudło, Paweł Krzyściak, Barbara Gryglewska, Anna Różańska, Jadwiga Wójkowska-Mach

**Affiliations:** 1 Department of Ophthalmology, St. Rose Hospital, Kraków, Poland; 2 Department of Microbiology, Faculty of Medicine, Jagiellonian University Medical College, Kraków, Poland; 3 Directorate-General for Health and Food Safety, European Commission, Brussels, Belgium; 4 Division of Pharmacoepidemiology and Clinical Pharmacology, Faculty of Science, Utrecht Institute for Pharmaceutical Sciences, Utrecht University, Utrecht, The Netherlands; 5 Department of Internal Medicine and Gerontology, Jagiellonian University Medical College, Kraków, Poland; 6 Department of Internal Medicine and Geriatrics, University Hospital, Kraków, Poland

**Keywords:** Antibiotics, Consumption, COVID-19, SARS-CoV-2, Healthcare-associated infection

## Abstract

**Background:**

COVID-19 has been challenging for the entire healthcare system, due to the lack of sufficient treatment protocols, especially during initial phases and as regards antibiotic use. The aim of this study was to identify the trends of antimicrobial consumption in one of the largest tertiary hospitals in Poland during COVID-19.

**Methods:**

This is a retrospective study conducted at the University Hospital in Krakow, Poland, between Feb/Mar 2020 and Feb 2021. It included 250 patients. All included patients were hospitalized due to COVID-19 with confirmed SARS-CoV-2 infection without bacterial co-infections during the first phase of COVID-19 in Europe and following 3-month intervals: five equal groups of patients in each. COVID severity and antibiotic consumption were assessed according to WHO recommendations.

**Results:**

In total 178 (71.2%) patients received antibiotics with a incidence rate of laboratory-confirmed healthcare-associated infection (LC-HAI) was 20%. The severity of COVID-19 was mild in 40.8%, moderate in 36.8%, and severe in 22.4% cases. The ABX administration was significantly higher for intensive care unit (ICU) patients (97.7% vs. 65.7%). Length of hospital stay was extended for patients with ABX (22.3 vs. 14.4 days). In total, 3 946.87 DDDs of ABXs were used, including 1512.63 DDDs in ICU, accounting for 780.94 and 2522.73 per 1000 hospital days, respectively. The median values of antibiotic DDD were greater among patients with severe COVID-19 than others (20.92). Patients admitted at the beginning of the pandemic (Feb/Mar, May 2020) had significantly greater values of median DDDs, respectively, 25.3 and 16.0 compared to those admitted in later (Aug, Nov 2020; Feb 2021), respectively, 11.0, 11.0 and 11.2, but the proportion of patients receiving ABX therapy was lower in Feb/Mar and May 2020 (62.0 and 48.0%), whereas the highest during the late period of the pandemic, i.e., in Aug, Nov. 2020 and Feb. 2021 (78% and both 84.0%).

**Conclusions:**

Data suggest great misuse of antibiotics without relevant data about HAIs. Almost all ICU patients received some antibiotics, which was correlated with prolonged hospitalization.

## Introduction

Coronavirus disease 2019 (COVID-19), which was initiated in Wuhan, Hubei Province of China, in December 2019, triggered a global pandemic with a high fatality rate [1]. Since the World Health Organization (WHO) declared the COVID-19 pandemic in March 2020, there have been over 627 million confirmed cases and over 6.5 million deaths worldwide as of October 30, 2022 [2]. COVID-19 is a highly contagious infection caused by the severe acute respiratory syndrome Coronavirus-2 (SARS-CoV-2), which mainly produces a series of respiratory symptoms [3]. Fortunately, most infected patients have an asymptomatic course or mild symptoms, but some of them develop severe respiratory distress, requiring hospitalization. In severe cases, patients require intensive care treatment and prolonged hospital stay [4] which are known factors that enhance the risk of developing healthcare associated, mostly bacterial, infection, and viral infections alone have been regarded as a factor that may contribute to developing bacterial co-infections [5].

Despite COVID-19 being a viral disease, the use of antimicrobials has been common in infected patients, especially at the beginning of the pandemic, due to the lack of treatment algorithms. A rapid review and meta-analysis of the studies published in the first six months of the pandemic revealed that 75% of patients with COVID-19 demonstrated high antibiotic consumption, despite the relatively low prevalence of co-infection and superinfections [6]. The meta-analysis by Khan et al. [7] of 19 studies measuring antimicrobial consumption as Daily Defined Doses (DDD) or days of therapy (DOT) or percentage, showed an overall high antimicrobial consumption of 68%. Consumption was lower in high-income countries compared to lower and middle-income countries (58% vs 89%). However, during the first few months of the COVID-19 pandemic, a substantial increase in antimicrobials was also observed in several high-income countries. Such high prescription of antibacterials was partly due to the difficulties posed by differentiating isolated severe COVID-19 cases from community-acquired pneumonia or COVID-19 with secondary bacterial infection [8]. The current WHO COVID-19 guidelines discourage the use of empirical antimicrobials in patients with mild or moderate COVID-19 (WHO COVID-19). Moreover, the guidelines advise rapid de-escalation of treatment in patients with a severe disease without confirmation of bacterial infection diagnosis.

The overuse of antibiotics influences antimicrobial resistance with the selection of multidrug-resistant organisms [9]. The high consumption of antibiotics also has a negative clinical impact with an elevation of morbidity (including healthcare-acquired *Clostridioides difficile* infections), mortality, and healthcare costs [9, 10]. Since antimicrobial use is one of the main drivers of antimicrobial resistance, surveillance, and optimization of antibiotic use are essential to keep it under control.

Reproduction of SARS-CoV-2 provides opportunities for the acquisition of mutations and different variants, altering viral transmissibility, disease severity, and some ability to escape from natural or vaccine-derived immunity [11]. SARS-CoV-2 variant B.1.1.7 (Alpha) and subsequent B.1.617.2 (Delta) were the dominant strains responsible for the generation of the first two phases of COVID-19. The present study aims to identify the trends of the antimicrobial consumption profile in one of the largest tertiary hospitals in Poland during the beginning of the COVID-19 pandemic between Feb/Mar 2020 and Feb 2021. Data recorded by the hospital have been analyzed to identify factors that were associated with antimicrobial consumption and illustrate how the situation was evolving over time during the first year of the pandemic. The secondary objective is to identify the determinants of antimicrobial use in studied hospital during the beginning of COVID-19 pandemic.

## Methods and materials

### Study design and study population

It was a retrospective study conducted at the University Hospital in Krakow in Poland (UHK), between Feb/Mar 2020 and Feb 2021. In 2020, there were 30,043 hospitalizations (excluding the Hospital Emergency Department). Between March and September 2020, UHK was designated to treat only COVID-19 patients. Patients were diagnosed with COVID-19 according to WHO guidelines using the RT-PCR method for SARS-CoV-2 [12].

The analysis was carried out on the healthcare data electronically registered by UHK and the hospital pharmacy. We included 250 adult patients hospitalized due to COVID-19 with confirmed SARS-CoV-2 infection without bacterial co-infections at admission during the first phase of COVID-19 infections in Europe (the baseline) and at subsequent 3-month intervals as follows:

- The baseline of the analysis was the beginning of the hospitalization of the first officially reported COVID-19 patient in UHK, which started even before the pandemic reached Poland–on Feb 13, 2020. Among the first 50 patients included in the analysis, there were also patients who suffered from COVID-19 infection during hospitalization (hospital-acquired COVID-19, HA-COVID-19).
- Next, 50 consecutive patients were admitted to the UHK on May 13, 2020, Aug 13, 2020, Nov 13, 2020, and Feb 13, 2021. The early phase was defined as March–August 2020 and the late phase was November 2020–February 2021. Patients with HA-COVID-19 were eligible for inclusion in this study.

All patients included in the analysis had confirmed SARS-COV-2 infection at the time of admission. Pre-hospitalization data were very sparse. Co-infections were excluded by a retrospective review of a given patient’s and department’s medical records, both electronic and on paper (the available laboratory tests and the description of the patient's condition from the daily physical examination, ward and patient documentation). Positive results of microbiological tests on the 3rd day of admission were a certain indication for the use of an antibiotic. However, in some clinical situations (clinical deterioration, large increase in calcitonin concentration, sputum collection difficulties, suspected bacterial sepsis), delaying antibiotic therapy may lead to increased mortality, and initial broad-spectrum empiric antibiotic therapy should be initiated. We did not analyze such data in our population. All antibiotics have some specific adverse side effects such as allergy or dose-related haematological, gastrointestinal, renal or hepatic side effects. However, we did not analyze such data and their association with patients’ outcome, except for *Clostridioides difficile* infections.

### COVID-19 disease severity classification

The course of COVID-19 was assessed on the basis of the WHO recommendations “Living guidance for clinical management of COVID-19” [https://www.who.int/publications/i/item/WHO-2019-nCoV-clinical-2021-2] according to which the severity of the course is classified as mild, moderate, severe, or critical—as ARDS (acute respiratory distress syndrome), septic shock, sepsis, acute thrombosis. In the present analysis, patients meeting the severe or critical criteria were included in one group: severe.

Patients who had material collected for microbiological testing during hospitalization, but not earlier than on the third day of admission and were positive for bacterial (mainly phenotypic methods and rapid membrane enzyme immunoassay test for *Clostridioides difficile*) were classified as patients with laboratory-confirmed healthcare-associated bacterial infections (LC-HAI). In the years 2020–2021, the UHK did not conduct active, prospective surveillance and registration of infections, therefore, there is no other source of information on nosocomial infections that were not microbiologically confirmed. The length of hospital stay of patients (LOS) was defined as the sum of days of stay, including the day of admission and the day of discharge.

### Antibiotic and glucocorticoid consumption measure

To assess the use of antibiotics, the WHO ATC-DDD system was adopted, including in particular: that the analyses were based on drugs from the J01 group, and the quantitative analysis used defined daily doses–values available on the ATC WHO DDD website. The average consumption per patient was employed to compare the use of antibiotics as a dependent variable depending on the selected factors.

The WHO ATC-DDD system was also adopted to evaluate the use of glucocorticoids as an independent variable group H01-02.

### Statistical analysis

For statistical computing, we used R version 4.2.2 (2022-10-31) with the RStudio environment (v 2022.07.1) and working libraries: dplyr (v. 1.0.7), questionr (v. 0.7.5), and oddsratio (v. 2.0.1). We compared the data in the group of patients treated with antibiotics and those without such treatment. Due to inconsistency with the normal distribution of the analyzed data, the Mann–Whitney or Kruskal–Wallis tests were applied. We used the Chi-square test to determine the significance of differences in the contingency tables, and the odds ratio was determined using Fisher’s exact test. In all cases, those at p ≤ 0.05 were considered statistically significant. To analyze which factors influence the use of antibiotic (dependent categorical variable), we build the logistic regression model including age (continuous), sex (categorical), ICU stays (categorical), LOS (continuous) and COVID severity. We did not find any significant interaction between ICU stays and COVID severity so it was omitted in this model.

## Results

In total, 178 (71.2%) patients received antibiotics (ABX) and the incidence rate of laboratory-confirmed healthcare-associated infection (LC-HAI) was 20%, including 2 cases of *Clostridioides difficile* infection. No patients had laboratory-confirmed bacterial co-infection at admission. Co-infections were excluded by a retrospective review of a given patient’s and department’s medical records, both electronic and on paper.

The severity of COVID-19 was mild in 102 (40.8%), moderate in 92 (36.8%), and severe in 56 (22.4%) patients, including 5 cases of septic shock and 1 case of laboratory confirmed bloodstream infection. Among patients with mild/moderate COVID, patient mortality was incidental during the study period and was 1.5% (*n* = 1) in patients without antibiotics and 3.2% (*n* = 4) in patients with antibiotic therapy, respectively. In the group with severe COVID, mortality was 71.0% (*n* = 10) in patients without antibiotics and 48.6% (*n* = 18) in patients with antibiotic therapy, respectively. The data indicate the validity of the therapy among patients with a severe course, which may be associated with a higher predilection for bacterial co-infections in this group of patients. The case fatality rate was 14.8% (Table 1). Forty-three (17.2%) patients were hospitalized in the intensive care unit (ICU), almost all patients (97.7%) admitted to the ICU were treated with antimicrobials. The use of antibiotics was significantly lower (*p* < 0.001) among non-ICU patients (65.7%), but it was still high.

**Table 1:** Characteristics of patients with and without antibiotic treatment

Characteristic	Patients with ABX, *N* (%)	Patients without ABX, *N* (%)	*p*-value
Number of patients	178 (71.2)	72 (28.8)	–
Age, mean (SD) [years]	64.7 (13.3)	51.7 (17.4)	Mann–Whitney *U* = 3509, *p* < 0.001
LOS, mean (SD) [days]	22.3 (16.3)	14.4 (10.2)	Mann–Whitney *U* = 4001, *p* < 0.001
ICU LOS*, mean (SD) [days]	3.2 (8.43)	0.4 (3.1)	Mann–Whitney *U* = 27.5, *p* = 0.6285
Sex			
Female	77 (67.5)	34 (29.8)	*χ*^2^ = 0.32627, df = 1, *p* = 0.5679
Male	101 (72.7)	38 (27.3)	
COVID severity			
Mild	46 (45.1)	56 (54.9)	*χ*^2^ = 57.838, df = 2, *p* < 0.001
Moderate	80 (87.3)	12 (13.0)	
Severe	52 (92.9)	4 (7.1)	
ICU			
Yes	42 (23.6)	1 (1.4)	*χ*^2^ = 17.751, df = 1, *p* < 0.001
No	136 (76.4)	71 (98.6)	
Death			
Yes	34 (19.1)	3 (4.2)	*χ*^2^ = 9.0676, df = 1, *p* < 0.0026
No	144 (80.9)	69 (95.8)	
GCS			
Yes	111 (62.4)	6 (8.3)	*χ*^2^ = 60.099, df = 1, *p* < 0.001
No	67 (37.6)	66 (91.7)	
Date of admission			
Feb 2020	31 (62.0)	19 (38.0)	*χ*^2^ = 24.306, df = 4, *p* < 0.001
May 2020	24 (48.0)	26 (52.0)	
Aug 2020	39 (78.0)	11 (22.0)	
Nov 2020	42 (84.0)	8 (16.0)	
Feb 2021	42 (84.0)	8 (16.0)	
LC-HAI			
Yes	50 (28.1)	0 (0.0)	*χ*^2^ = 25.281, df = 1, *p* < 0.001
No	128 (71.9)	72 (100.0)	

*Only ICU patients; *GCS* glucocorticoids, *ICU* intensive care unit, *LC-HAI* laboratory-confirmed healthcare-associated infections, *LOS* length of stay, *SD* standard deviation

The study was conducted at the University Hospital in Krakow, Poland, between Feb/Mar 2020 and Feb 2021

Patients treated with ABX were significantly older (mean age 64.7 ± 13.3 years) than those without such medicines (51.7 ± 17.4 years). As we expected, the use of antimicrobials was significantly less frequent among patients with mild severity of COVID-19, compared to those with moderate or severe course of the disease (respectively, 45.1 vs 87.3 vs 92.9%).

Length of stay was higher among patients receiving ABX than those without such treatment (on average 22.3 compared to 14.4 days, respectively). The distribution of patients receiving antibiotic therapy changed throughout the time periods covered. The lowest proportion was recorded in May 2020 (48.0%), whereas the highest was observed during the late period of the pandemic, i.e., in Nov. 2020 and Feb. 2021 (both 84.0%) (Tables 1, 2). The systemic GCS were used in 117 patients (46.8% of all); significantly more often in patients with ABX (*p* < 0.001).

**Table 2:** Comparison of the early and late phases of the pandemic groups

Characteristic	Phases of the pandemic *N* (%)		*p*-value
	Early phase	Late phase	
Age, mean (SD) [years]	54.9 (15.8)	65 (14.3)	Mann–Whitney *U* = 4689.5, *p* < 0.0001
LOS, mean (SD) [days]	22.7 (17.4)	18.2 (13.3)	Mann–Whitney *U* = 8650.5, *p* = 0.0399
ICU LOS, mean (SD)* [days]	13.23 (13.44)	14.42 (12.36)	Mann–Whitney *U* = 342, *p* = 0.002743
Sex			
Female	50 (50.0%)	61 (40.7%)	χ^2^ = 2.1172, df = 1, *p* = 0.1457
Male	50 (50.0%)	89 (59.3%)	
ABX			
Yes	55 (55.0%)	123 (82.0%)	*χ*^2^ = 21.331, df = 1, *p* < 0.0001
No	45 (45.0%)	27 (18.0%)	
COVID severity			
Mild	67 (67.0%)	35 (23.3%)	*χ*^2^ = 48.123, df = 2, *p* < 0.0001
Moderate	18 (18.0%)	74 (49.3%)	
Severe	15 (15.0%)	41 (27.3%)	
ICU			
Yes	17 (17.0%)	26 (17.3%)	*χ*^2^ = 0.0046811, df = 1, *p* = 0.9455
No	83 (83.0%)	124 (82.7%)	
Death			
Yes	10 (10.0%)	27 (18.0%)	*χ*^2^ = 3.0453, df = 1, *p* = 0.0809
No	90 (90.0%)	123 (82.0%)	
GCS			
Yes	19 (19.0%)	52 (34.7%)	*χ*^2^ = 51.735, df = 1, *p* < 0.0001
No	81 (81.0%)	98 (65.3%)	
LC-HAI			
Yes	18 (18.0%)	32 (21.3%)	*χ*^2^ = 0.41667, df = 1, *p* = 0.5186
No	82 (82.0%)	118 (78.7%)	

^*^only for ICU patients

*ABX* antibiotics, *GCS* glucocorticoids, *ICU* intensive care unit, *LC-HAI* laboratory-confirmed healthcare-associated infections, *LOS* length of stay, *SD* standard deviation

The study was conducted at the University Hospital in Krakow, Poland, between Feb/Mar 2020 and Feb 2021

**Fig. 1:**
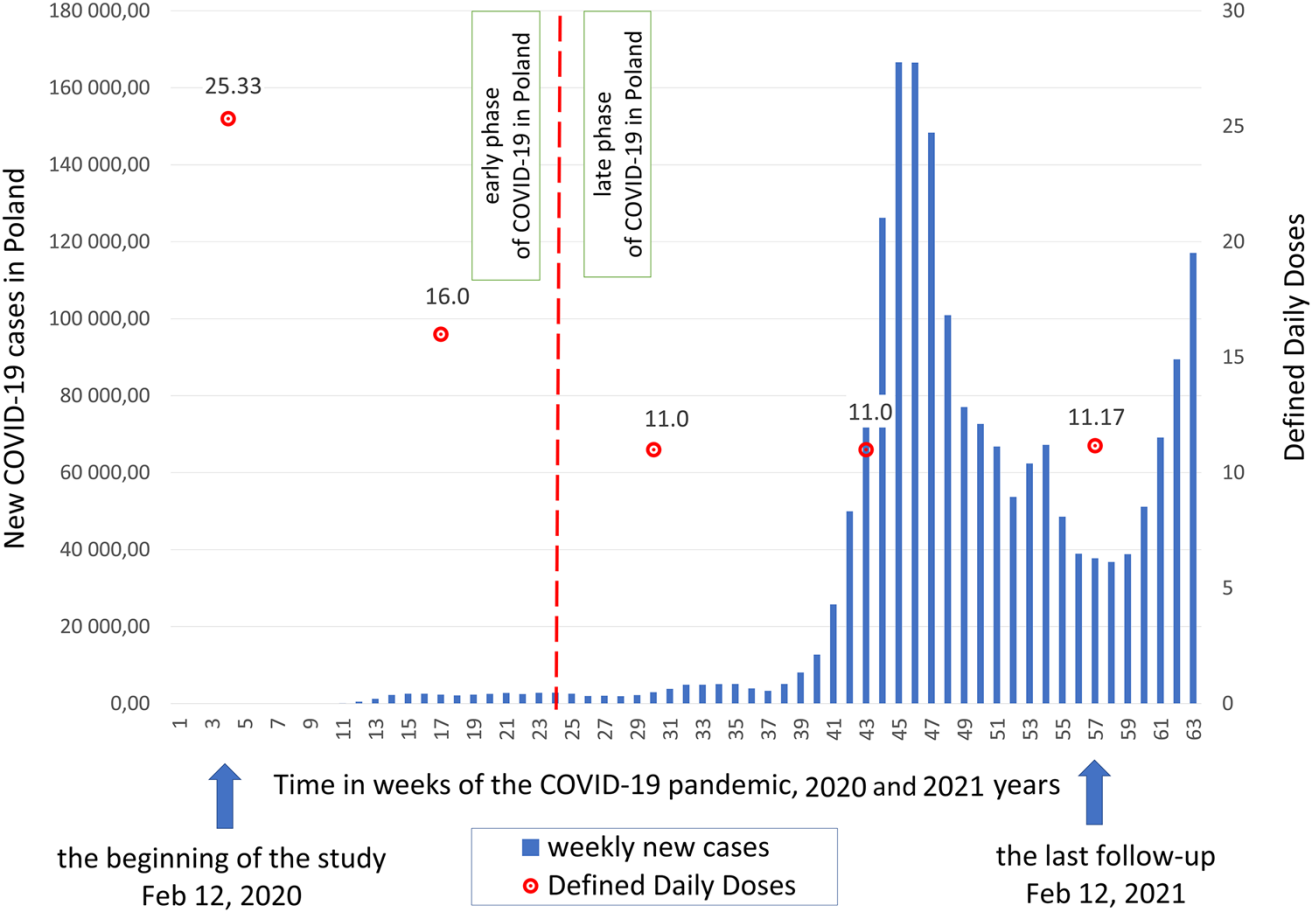
Defined Daily Doses and new cases of COVID-19 during the course of pandemic. The study was conducted at the University Hospital in Krakow, Poland, between Feb/Mar 2020 and Feb 2021

The hypothesis about significant trends of changes during all of the analyzed phases of the pandemic turned out to be irrelevant changes fluctuating between individual periods, while in the combined analysis of all periods, these trends blur. Only after stratification of data into 2 periods, before vs. during the high weekly incidence rate (March–August 2020 vs. Nov 2020–Feb 2021), significant differences were observed, both in the population of treated patients and in the intensity of treatment, both with ABX and GS (Fig. 1, Table 2).

In the whole study group, 3 946.87 DDDs (85.7 per 100 patient days, pds) of antibiotics were used, and among patients hospitalized in ICU the amount reached 1 512.6 DDDs (78.1/100 pds in non-ICU settings and 252.3/100 pds in ICU).

In 178 patients who did receive antibacterial medicinal products, the median values of antibiotic DDDs consumed were greater among males, 16.50 DDDs per patient, compared to 11.00 in females (Mann–Whitney test, *U* = 2846.5, *p* = 0.0022). Patients with severe COVID-19 had the highest values of antimicrobial consumption, the median of DDDs in this group amounted to 20.92 (*p* < 0.001). Patients admitted at the beginning of the pandemic (February, May 2020) had significantly greater values of median DDDs, respectively, 25.3 and 16.0, compared to those admitted in later months of the pandemic (August, November 2020; February 2021), respectively, 11.0, 11.0 and 11.2 (Fig. 1).

Higher consumption of ABX is reported in the ICU patients and with LC-HAIs. Significant differences concerning DDDs were not observed between groups of patients who were administered systemic GCS and those who did not receive this kind of therapy (Table 3). The GCS consumption was higher for patients with antibiotics (62.4% vs. 8.3%), but it does not influence the DDD of ABX (Mann–Whitney test, *U* = 3680.5, *p* = 0.91).

**Table 3:** Defined Daily Doses of antibiotics compared to different factors

Factors	DDD of ABX [Median (Q1; Q3)]	*p*-value
Sex		
Male	16.5 (10.0; 28.0)	Mann–Whitney *U* = 2846.5, *p* = 0.0022
Female	11.0 (10.0; 17.2)	
COVID-19 severity		
Mild	12.4 (8.0; 25.2)	Kruskal–Wallis, χ^2^ = 9.885, df = 2, = < 0.0001
Moderate	11.0 (8.0; 17.1)	
Severe	20.9 (12.0; 41.3)	
Date of admission		
Feb 2020	25.3 (16.1; 47.5)	Kruskal–Wallis, *χ*^2^ = 23.621, df = 2, *p* < 0.0001
May 2020	16.0 (12.8; 26.5)	
Aug 2020	11.0 (6.5; 21.5)	
Nov 2020	11.0 (8.0; 20.2)	
Feb 2021	11.2 (8.2; 14.8)	
ICU		
Yes	25.2 (14.6; 45.0)	Mann–Whitney *U* = 1492.5, *p* < 0.0001
No	11.2 (8.0; 19.2)	
LC-HAI		
Yes	24.2 (11.4; 46.3)	Mann–Whitney *U* = 1892.5, *p* < 0.0001
No	12.0 (8.00; 19.0)	
GCS		
Yes	12.0 (8.2; 29.0)	Mann–Whitney *U* = 3680.5, *p* = 0.9103
No	13.0 (9.0; 20.7)	

*DDD* defined daily doses, *GCS* glucocorticoids, *ICU* intensive care unit, *LC-HAI* laboratory-confirmed healthcare-associated infections, *Q1 and Q3* first quartile and third quartile

The study was conducted at the University Hospital in Krakow, Poland, between Feb/Mar 2020 and Feb 2021

Multivariable analysis shows that the following have a significant impact on antibiotic use: LOS (OR 1.03; 95%CI 1.004–1.07), age (OR 1.04; 95%CI 1.01–1.06), and COVID-19 severity (OR 3.79; 95%CI 2.10–7.12). The ICU presents a positive tendency to influence antibiotic use, but in our analysis, it was statistically insignificant (Table 4).

**Table 4:** Multivariable analysis of the influence of the studied factors on the antibiotic use, not significant factors were omitted

The studied factors	OR	95%CI	*p*-value
LOS	1.03	1.004–1.07	0.0438
Age	1.04	1.01–1.06	0.0032
ICU (yes)	5.80	1.05–108.60	0.0962
COVID severity	3.79	2.10–7.12	< 0.0001

*ICU* intensive care unit, *LOS* length of stay, *OR 95%CI* Odds ratio and 95% confidence interval

The study was conducted at the University Hospital in Krakow, Poland, between Feb/Mar 2020 and Feb 2021

## Discussion

In the studied population, ABX were used in over 70% of patients, significantly more often in the second period of the pandemic, when as many as 82% of patients received them. During the first phase, UHK was the only hospital in the region of Małopolska which almost all hospitalized patients with confirmed SARS-COV-2 infections stayed in, independently of their clinical status. Later, many regional COVID-19 hospitals were organized, and UHK admitted mainly severe cases or patients with clinical complications of COVID-19 who needed specialized treatment. These aspects had a significant impact on the antibiotic prescription rate.

On the other hand, in the first phase, the consumption of ABX was significantly more than 2 times higher and reached as many as 25 DDD, with a significantly higher proportion of patients with severe COVID-19. The retrospective study did not take into account the reasons behind the decisions made by clinicians at various stages of the pandemic. Those may have resulted from various factors, independent of one another. However, the aspect of paramount importance might have been the physicians’ anxiety associated with the new clinical situation. Clear criteria concerning the application of various drug groups, also antibiotics, appeared much later—during the second phase of our observation. In the beginning of the pandemic, doctors employed the medicines that were in line with their previous experiences—and they had no experience in treating COVID-19—and made use of their own risk assessment. Antimicrobial stewardship programs are needed to support evidence-based antibiotic prescribing decisions and can contribute to reducing unnecessary antibiotic use without adversely affecting patients. The optimal duration of antibiotic therapy depends on clinical symptoms and the causative organism. The current data suggest that there is no risk in stopping a course of an antibiotic immediately after exclusion of bacterial infection and there is no difference in outcome when shorter courses are used.

The reason for the problem of abuse of antibiotics in the beginning of the pandemic can also be the communication culture, which, according to Hofstede’s theory, is characterized by the high so-called “uncertainty avoidance" in Poland. Unfortunately, up to date—the end of 2022—there is only limited treatment for SARS-CoV-2 infection. The system focuses on symptomatic treatment, respiratory support and medication used in patients with severe COVID-19 in ICUs, which include anaesthetics or resuscitation medicines, as well as some antibiotics, also used off-label. In Europe, eight medicinal products for COVID-19 were authorized by the European Commission (information from September 2022) [13]. The first COVID-19 treatment in the EU was remdesivir [14]. The marketing authorization was granted in July 2020 for the treatment of COVID-19 in adults and adolescents from 12 years of age with pneumonia who require supplemental oxygen. It is noteworthy that, for several months, that was the only medicine authorized in the EU for COVID-19.

Several guidelines at European and international levels were published to support clinicians in their decisions. However, during the first phase of the pandemic and the first clinical decisions, there were no precise recommendations. Knowledge about the natural course of the disease was incomplete. The guidelines of various societies and organizations—including Polish ones—available in the first months were often based on the interpretation of scarce, partial data and pathophysiological considerations. Polish recommendations of the Polish Society of Epidemiologists and Infectiologists recommended that in critical or severe COVID-19 cases (e.g., ARDS) antibiotic therapy should be implemented because it is difficult to clearly exclude the coexistence of bacterial infection. But even in these patients, it should not be a routine procedure [15]. The ECDC, in its publication *Treatment and pharmaceutical prophylaxis of Covid-19,* recommends a conscious use of antibiotics in patients with COVID-19 [16].

According to the ECDC report from 2021, the total antibiotic consumption in humans in most EU/EEA countries decreased by 15% between 2019 and 2020 [17]. However, despite the viral etiology of the disease, many countries have seen a significant increase in the consumption of antibiotics in the early phase of the pandemic. It was the case, e.g., in the United Kingdom [7], United States [18], Brazil [19], Portugal [20], or Iran [21]. This resulted from, on one hand, wanting to reduce the risk of bacterial coinfections and their complications, including death of patients, but on the other hand, the lack of guidelines or the possibility of targeted treatment.

Every year, the epidemiological report on antimicrobial consumption is published by the ECDC. Certainly, monitoring is one of the core elements of antimicrobial stewardship [22]. However, an outbreak of a novel viral respiratory disease caused by SARS-CoV-2 was associated with a considerable risk of hospitalization and death. According to the ECDC data, case fatality rate of COVID-19 in the whole world population in 2020 was 2.3% [23], whereas in a study from the Ochsner Center for Outcomes and Health Services Research, Louisiana, USA, in-hospital case fatality rate at the beginning of the pandemic was 8.9% [24], which was similar to the one in Poland at 8.0% [25]. And in that situation, a sudden change in the functioning of healthcare facilities was needed. It required accelerated adaptation to accommodate the increasing number of patients with COVID-19. The unprecedented circumstances may have impacted the way antimicrobial agents were used.

In our study, in-hospital mortality in the first phase of the pandemic was similar to the one described by other authors [24, 25], but unfortunately, it rose from 10 to 18% in the second phase of the pandemic, when the share of patients with severe COVID-19 increased significantly. However, as the situation worsened—raising the share of patients in serious condition—the antibiotic consumption was halved, from 25.33 DDD/per patient at the beginning to 11 DDD/per patient. Interestingly, at the same time, the proportion of patients receiving antibiotics increased significantly to 82%. This was also observed in Pakistan (patients prescribed antibiotics, 89.7%), where bacterial co-infection or secondary infection were confirmed very rarely, in only 1.1 and 3.1% of patients, respectively [26].

Despite the fact that GCS administration was higher for patients with antibiotics (62.4% vs. 8.3%), this does not influence the DDD of antibiotics. The WHO Rapid Evidence Appraisal for COVID-19 Therapies (REACT) Working Group, in their meta-analysis, point out that in severe COVID-19, GCS did not have such a negative effect on HAIs or mortality as in influenza or other viral infections [27]. Most of the studies show a reduction in mortality due to decreasing the inflammatory changes described in autopsy examination [28, 29]. In Poland, oral GCS were used for patients with hypoxia or with at least mild respiratory symptoms, for which most of them also got antibiotics. This may explain why DDD was not increasing as in other viral infections.

In our study, the model of multivariable analysis revealed that ICU hospitalization increased the risk of antibiotic use almost 6 times and COVID severity almost 4 times. Age of patients and length of hospital stay increased that risk by only about several percent. Probably, the LOS was interrelated with other clinical aspects of hospitalization, age of the patient and also the antibiotic use. At the same time, we observed an increase in antibiotic prescription (not antibiotic consumption expressed in DDD) later during the pandemic and at the same time, a strong relationship of severity of COVID-19 and case fatality rate. It is difficult to clearly explain this fact, but in accordance with the guidelines for the treatment of SARS-COV-2 infection, patients with the mild course of the disease should be treated only symptomatically—the deterioration of the patient’s condition and increase of inflammatory markers levels may suggest a possible co-infection and indications of antibiotic prescription [16], that may explain the interdependence between COVID-19 severity, antibiotic prescription, and prognosis.

The observed incidence of LC-HAI did not change during the 12-month follow-up and was very high, 20%, despite the fact that only 1/5 of the patients were hospitalized in the ICU. According to a multicenter, international, observational study by Conway Morris et al. the bacterial HAI incidence in COVID-19 patients was 54%, but only in ICU patients [30]. The very high rate of LC-HAI observed in our study may explain the total level of antibiotics used per 1000 hospital days, which was 780.94, while Bitterman et al. in a systemic review based on 80 studies, observed an average value of 586 DDD/1000 hospital days, ranging from 540 to 632 [31]. Also for the ICU, the respective value in our study was about three times higher than the one in the multicenter study done by Barnsteiner et al. covering 58 ICUs in Switzerland over a ten-year period (2009–2018) [32]. This high level of antibiotic consumption rather cannot be explained by the general pandemic situation, because the ESAC report for the year 2020 shows that overall antibiotic consumption in the Polish hospital sector was lower in the year 2020 compared to the year 2019 [33], hence our hypothesis of potentially irrational antibiotic therapy.

## Limitations

Due to the retrospective nature of the study, it was based on medical records. Due to the limitations concerning the personnel working in full personal protective equipment, some inaccuracies are possible due to missing or incomplete patient records. Extreme outlier results were critically assessed and then qualified or rejected from the analysis by the team of authors depending on the likelihood of such a clinical situation or the risk of an incorrect entry. The retrospective nature of the study makes it impossible to clearly exclude bacterial co-infection on admission to the hospital, however, taking into account the available laboratory tests and the description of the patient’s condition from the daily physical examination (ward and patient documentation), co-infections at admission were assessed as unlikely.

Next, the positive microbiological tests were used in our study to confirm HAIs and the necessity of antibiotic use. Although the consideration of antibiotic treatment in critically ill patients with COVID-19 should be advised as the bacterial infection cannot be excluded, such clinical decision should be associated with microbial tests, especially as it is difficult to distinguish bacterial or fungal infections from existing viral pneumonia based on clinical and radiological findings. And finally, due to the lack of data on whether patients were prescribed antibiotic treatment for the pre-admission and post-discharge periods, it is not possible to determine the degree of dependence of the studied variables mentioned, however, the variables are not interrelated and antibiotic treatment prolongs the patient's stay in the hospital. Nevertheless, the trend of inpatient care tends towards rapid discharge of patients to continue treatment in outpatient settings, if the patient's general condition allows for safe discharge of the patient.

## Conclusions

The study found that antibiotic therapy was very common in COVID-19 patients during the first phase in Poland. Almost all ICU patients received an antibiotic during their hospitalization, but the highest prescription of antibiotics was observed during the second period of the study, which was the first significant peak of COVID-19 cases in Poland. Thus, the results of our study show not only the lack of clear, consistent guidelines for antibiotic therapy in the first year of the pandemic but also a problem with the broader approach to antibiotic stewardship in Poland.

## Data Availability

The datasets generated or analyzed during this study are available and can be obtained, at request, from Jadwiga Wójkowska-Mach (e-mail: jadwiga.wojkowska-mach@uj.edu.pl) on reasonable enquiry.

## List of abbreviations

ABX: Antibiotics
ARDS: Acute respiratory distress syndrome
ATC: Anatomical Therapeutic Chemical
COVID-19: Coronavirus disease 2019
DDD: Daily defined doses
DOT: Days of therapy
ECDC: European Centre for Disease Prevention and Control
HAI: Healthcare-associated infection
ICU: Intensive care unit
LC-HAI: Laboratory-confirmed healthcare-associated infection
OR 95%CI: Odds ratio and 95% confidence interval
RT-PCR: Real-time polymerase chain reaction
SARS-CoV-2: Severe acute respiratory syndrome Coronavirus-2
UHK: University Hospital in Krakow, Poland
WHO: World Health Organization
